# Disseminated Intravascular Coagulopathy Caused by Uterine Leiomyoma with Sarcoma-Like Findings on Magnetic Resonance Imaging

**DOI:** 10.1155/2014/978743

**Published:** 2014-07-13

**Authors:** Akiyo Taneichi, Hiroyuki Fujiwara, Yukako Mizoguchi, Shizuo Machida, Hiroaki Nonaka, Yuji Takei, Yasushi Saga, Mitsuaki Suzuki

**Affiliations:** Department of Obstetrics and Gynecology, School of Medicine, Jichi Medical University, 3311-1 Yakushiji, Shimotsuke, Tochigi 329-0498, Japan

## Abstract

A leiomyoma rarely causes disseminated intravascular coagulopathy (DIC). In the present report, we describe a case of DIC caused by leiomyoma. A 36-year-old nulliparous woman presented with hypermenorrhea and a lower abdominal mass. On magnetic resonance imaging, we detected a 14 cm uterine tumor, which was suspected to be a sarcoma. Blood tests at the preoperative examination indicated platelet count of 9.6 × 10^4^/*μ*L, fibrin degradation product level of 107.1 *μ*g/mL (normal value, 0–5.0 *μ*g/mL), and fibrinogen level of 54 mg/dL (normal value, 129–271 mg/dL). Based on these findings, we diagnosed the patient with DIC. The patient was treated with nafamostat mesilate and fresh frozen plasma, but the DIC did not show any improvement. Subsequently, a hysterectomy was performed, after which the DIC improved. Clinicopathological findings indicated the presence of a leiomyoma with multiple vessels containing thromboemboli, and suggested that the DIC was caused by the leiomyoma. Therefore, it is essential to consider that that a benign leiomyoma may be a cause of DIC.

## 1. Introduction

Leiomyoma occurs in 30–40% of women and is one of the most common benign gynecological diseases. Leiomyoma may present with a variety of symptoms, such as hypermenorrhea and dysmenorrhea. Furthermore, an advanced malignant disease may reportedly coexist with disseminated intravascular coagulopathy (DIC). However, cases wherein a leiomyoma has caused DIC are very rare. Magnetic resonance imaging (MRI) is useful for distinguishing between leiomyoma and sarcoma. In the present report, we describe a case wherein the tumor was suspected as sarcoma on MRI but was subsequently identified as a leiomyoma that also caused DIC.

## 2. Case Presentation

**Figure 1:**
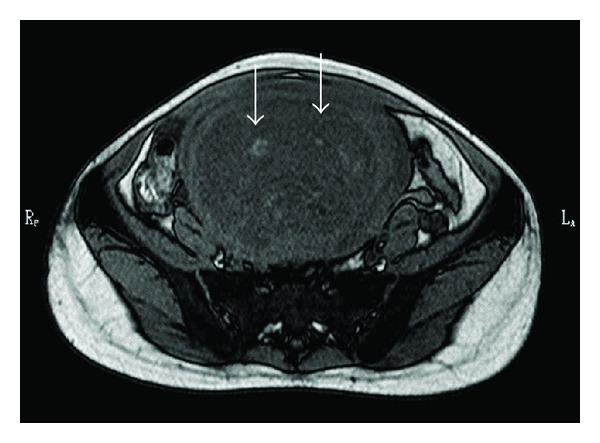
Magnetic resonance imaging of the uterine tumor showing a small area of high-signal intensity (arrows) on a T1-weighted image.

**Figure 2:**
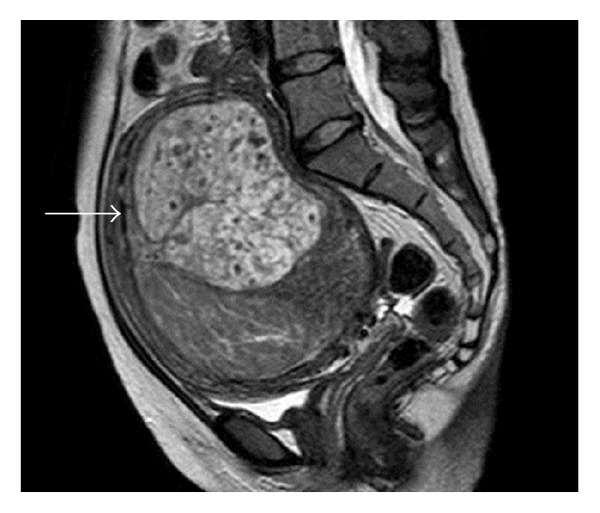
Magnetic resonance imaging of the uterine tumor showing heterogeneous high-signal intensity (arrows) on a T2-weighted image.

**Figure 3:**
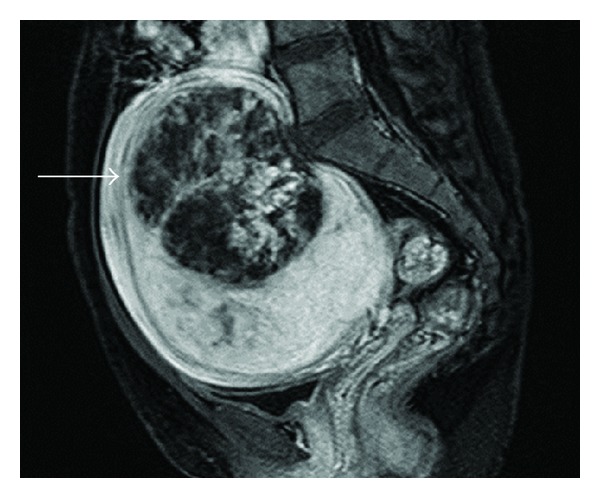
Magnetic resonance imaging of the uterine tumor showing heterogeneous contrast enhancement (arrows).

A 36-year-old nulliparous woman presented with a 4-5-year history of hypermenorrhea. The woman visited a clinic because of the presence of a palpable growing mass in her lower abdomen over several months. She presented with a uterine tumor of approximately 10 cm in diameter and anemia (hemoglobin (Hb) level: 4.7 g/dL) and was treated for the anemia with iron supplements. The woman was referred to our hospital for investigating the growing tumor. She had no atypical genital bleeding, except for hypermenorrhea. Physical examination indicated a mobile, firm, nontender solid mass arising in the pelvis and extending to below the umbilicus, by 1 fingerbreadth. Furthermore, vaginal sonography revealed a uterine tumor, 10 cm in diameter, which appeared as a uterine leiomyoma and had anonymous features. She had no remarkable medical history, had never used oral contraceptives, and had never received a blood transfusion. Moreover, she did not have any family history of thromboembolisms. We performed pelvic MRI with and without gadolinium contrast enhancement. On MRI, we identified a 14 cm uterine tumor in the pelvis. The uterine tumor grew from 10 cm to 14 cm within 1.5 months from the first visit. Furthermore, MRI indicated the presence of the uterine tumor with a small section of high-signal intensity on T1-weighted images (T1WI) (Figure 1), a heterogeneous high-signal intensity on T2-weighted images (T2WI) (Figure 2), and heterogeneous contrast enhancement (Figure 3). Thus, based on the growth of the uterine tumor and the MRI findings, we suspected the presence of a malignant uterine tumor, such as a uterine sarcoma; therefore, we decided that the patient should undergo a total hysterectomy. However, the levels of tumor markers were not elevated cancer antigen 125 (CA125), 28 U/mL (normal level, <35 U/mL), or cancer antigen 19-9 (CA19-9), 17 U/mL (normal level, <36 U/mL). Moreover, the lactate dehydrogenase (LDH) level was slightly elevated (226 mU/mL; normal value, 109–216 mU/mL). A preoperative examination indicated a prolonged blood coagulation time of 4.5 min (normal time, 0.5–3.0 min). The blood tests were repeated and confirmed the prolonged coagulation time (5 min) and thrombocytopenia. The biochemical data are shown in Table 1. Briefly, the white blood cell count (WBC) was 7000/*μ*L, Hb level was 11.7 g/dL, and platelet count was 9.6 × 10^4^/*μ*L, whereas the prothrombin time and activated partial thromboplastin time were normal. However, the thrombin/antithrombin III complex (TAT) level was elevated to 46.3 ng/mL (normal level, <2.4 ng/mL), indicating that the blood coagulation system was activated. Moreover, the fibrin degradation product (FDP) level was elevated to 107.1 *μ*g/mL (normal value, 0–5.0 *μ*g/mL), D-dimer level was elevated to 37.4 *μ*g/mL (normal value, 0–1.5 *μ*g/mL), plasmin *α*2-plasmin inhibitor complex (PIC) level was increased to 6.4 *μ*g/mL (normal level, <0.9 *μ*g/mL), and fibrinogen level was decreased to 54 mg/dL (normal value, 129–271 mg/dL), indicating that the fibrinolysis system was also activated. These data suggest that DIC was present. Moreover, we performed thoracic and abdominal computerized tomography (CT) and blood examination for further diagnostic analysis; however, we did not identify any other disease causing the DIC, such as sepsis or malignant tumors other than uterine tumors. Thus, we suspected that the cause of the DIC was the uterine tumor.

**Figure 4:**
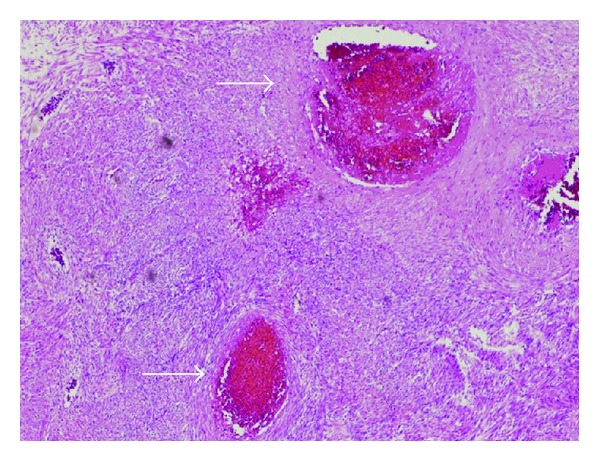
Microscopic view showing necrosis caused by circulatory disturbance because of multiple infarctions (arrows) in the tumor (hematoxylin-eosin stain ×40 magnification).

The woman was hospitalized and treated with 200 mg/day nafamostat mesilate [1] and transfused with 4 units of fresh frozen plasma (FFP). However, thrombocytopenia and hypofibrinogenemia were still noted, and the DIC did not improve. Seven days after admission, we performed a total abdominal hysterectomy. We transfused 4 units of FFP during this operation. The extracted uterus weighed 1200 g and was soft and pale yellow. On gross examination, the uterine tumor was 14 cm in diameter and some parts of the tumor showed degeneration, hemorrhage, and sponge-like cystic changes containing blood. Microscopic examination showed interlacing spindle-shaped smooth muscle cells with 5 mitotic figures/10 high-power fields and without remarkable nuclear atypia. We observed several vessels with thromboemboli and necrosis caused by circulatory disturbance in the tumor (Figure 4). The patient was finally diagnosed with leiomyoma and DIC caused by multiple vessels containing thromboemboli in the leiomyoma. The DIC improved 3 days after the operation (data shown in Table 1), and the patient was discharged 9 days after the operation. We subsequently investigated whether the patient had collagen disease or other thrombogenic factors but did not identify any such factors.

## 3. Discussion

In the present report, we describe the case of a patient with DIC caused by leiomyoma. MRI suggested that the uterine mass was a sarcoma. The high blood flow, degeneration, bleeding, and necrosis caused by the multiple thrombi within the leiomyoma resulted in a sarcoma-like appearance on MRI. To our knowledge, this is the first report of a uterine leiomyoma causing DIC with specific MRI findings.

In the present case, it is clear that the leiomyoma caused the DIC, as the DIC improved following the surgical removal of the uterus containing the tumor. The many causes of DIC include sepsis, severe burns, and placental abruption. Certain reports have indicated that malignant diseases may cause DIC; however, very few studies have investigated the link between benign disease, especially leiomyoma, and DIC. Four cases of leiomyoma accompanied by DIC have been reported in the English literature. Two cases were identified with degeneration of the leiomyoma during pregnancy [2, 3], and leiomyoma without pregnancy was the cause of DIC in the other two cases [4, 5]. Multiple thromboemboli were found within the leiomyoma, and the DIC improved following surgical removal of the leiomyoma in all cases. As the leiomyoma in the present case contained several vessels, it was necessary to distinguish between leiomyoma and angioleiomyoma of the uterus. Angioleiomyoma is a benign tumor that most often occurs in the lower extremities. Approximately 15 cases of uterine angioleiomyoma have been reported [6, 7], including a case with consumptive coagulopathy [7]. The characteristic features of angioleiomyoma include the presence of bland, spindle-shaped smooth-muscle cells, numerous thick-walled arteriole-like vessels, and swirling of the smooth-muscle cells around the vessels. The patient in the present case did not have many vessels or swirling of the smooth-muscle cells around the vessels. Moreover, angioleiomyoma has not been classified as a variant of leiomyoma in the World Health Organization classification of tumors of the female genital organs. Hence, we diagnosed the tumor as a usual leiomyoma with prominent vessels.

Leiomyoma is a benign disease, and it is important to distinguish this disease from malignant diseases such as sarcoma. The clinical symptoms of rapid growth and MRI findings are useful for the differential diagnosis between leiomyoma and sarcoma. In the present case, the uterine tumor demonstrated growth and appeared to be a sarcoma on an MRI scan. A typical leiomyoma shows an iso/low-intensity on T1WI and a low-intensity on T2WI on MRI. In contrast, sarcomas show high-intensity on T1WI, high-intensity on T2WI, and heterogeneous contrast enhancement. One study compared the MRI findings of 24 patients with either smooth muscle tumors of uncertain malignancy (SMTUMP), leiomyosarcoma, or leiomyoma clinically suspected as sarcoma [8]. They reported that there was a high possibility that the tumor was not a leiomyoma when it showed high-intensity on T1WI and high-intensity on T2WI with a well-demarcated unenhanced area. On MRI, a high-intensity signal on T1WI is caused by the presence of intratumoral hemorrhage within the necrotic foci; many sarcomas have necrosis and hemorrhage in a local area. Moreover, a strong contrast effect is observed due to the hypervascularity of sarcomas. The present case demonstrated a high-contrast image due to the presence of abundant vessels and demonstrated a high-intensity area on T1WI and T2WI because of the degeneration and hemorrhage in the tumor and necrosis due to intratumoral infarction. These characteristics were responsible for the sarcoma-like MRI findings observed in the present case.

We used nafamostat mesilate and FFP to treat DIC, but it did not show any improvement. Nafamostat mesilate is a synthetic serine protease inhibitor and is commonly used in Japan for the treatment of DIC [1]. Nafamostat mesilate inhibits activated factors VIIa, Xa, and XIIa, thrombin kallikrein, plasminogen activator, and plasmin and has both anticoagulant and antifibrinolytic effects [1]. As the TAT and PIC levels were elevated, which indicated that the blood coagulation system and fibrinolysis system were activated, we treated the patient with nafamostat mesilate. However, the gold standard of the treatment of DIC involves the resolution of the underlying cause. In the present case, DIC improved within three days after the total abdominal hysterectomy.

We discussed an extremely rare case of DIC caused by leiomyoma, a common benign gynecological disease. In the present case, DIC developed because of the presence of multiple thromboemboli within the leiomyoma. The presence of necrosis, degeneration and hemorrhage due to intratumoral infarction, and multiple vessels containing thromboemboli of the leiomyoma resulted in a sarcoma-like appearance on MRI. In cases where sarcoma is suspected, based on the clinical symptoms and MRI findings, it is necessary to perform surgery and histopathological analysis for accurate diagnosis and treatment. Therefore, it is important to consider that benign leiomyoma can potentially cause DIC.

## Figures and Tables

**Table 1:** Preoperative and postoperative examination data.

		Normal value	Preoperatively			Three days postoperatively
WBC	(/*μ*L)	3500–9100		7000		5900
Hb	(g/dL)	11.3–15.2		11.7		13.1
plt	(×10^4^/*μ*L)	13.0–36.9		9.6	↓	21.7
PT	(s)	10.4–12.2		12.9		11.7
PT-INR		0.9–1.2		1.11		1.02
APTT	(s)	23.1–36.3		33.2		28.8
TAT	(ng/mL)	<2.4		46.3	↑	—
FDP	(*μ*g/mL)	0–5.0		107.1	↑	4.8
D-dimer	(*μ*g/mL)	0–1.5		37.4	↑	1.6
PIC	(*μ*g/mL)	<0.9		6.4	↑	—
Fibrinogen	(mg/dL)	129–271		54	↓	390

WBC: white blood cell count.

Hb: hemoglobin level.

plt: platelet count.

PT: prothrombin time.

PT-INR: prothrombin time-international normalized ratio.

APTT: activated partial thromboplastin time.

TAT: thrombin/antithrombin III complex.

FDP: fibrin degradation product.

PIC: plasmin *α*2-plasmin inhibitor complex.
